# GLP-1a: Going beyond Traditional Use

**DOI:** 10.3390/ijms23020739

**Published:** 2022-01-10

**Authors:** Lucas Fornari Laurindo, Sandra Maria Barbalho, Elen Landgraf Guiguer, Maricelma da Silva Soares de Souza, Gabriela Achete de Souza, Thiago Marques Fidalgo, Adriano Cressoni Araújo, Heron F. de Souza Gonzaga, Daniel de Bortoli Teixeira, Thais de Oliveira Silva Ullmann, Katia Portero Sloan, Lance Alan Sloan

**Affiliations:** 1 Department of Biochemistry and Pharmacology, School of Medicine, University of Marília, Avenida Higino Muzzi Filho, Marília 17525-902, SP, Brazil; lucasffffor@gmail.com (L.F.L.); elguiguer@gmail.com (E.L.G.); maricelma.soares.souza@gmail.com (M.d.S.S.d.S.); gabriela.achete@outlook.com (G.A.d.S.); adrianocressoniaraujo@yahoo.com.br (A.C.A.); herongonzaga@yahoo.com.br (H.F.d.S.G.); tholiveira011@gmail.com (T.d.O.S.U.); 2 Postgraduate Program in Structural and Functional Interactions in Rehabilitation, University of Marília, Marília 17525-902, SP, Brazil; 3 Department of Biochemistry and Nutrition, School of Food and Technology of Marilia (FATEC), Marília 17500-000, SP, Brazil; 4 Department of Psychiatry, Federal University of São Paulo, R. Sena Madureira 04021-001, SP, Brazil; marquesfidalgo@yahoo.com.br; 5 Postgraduate Program in Animal Health, Production and Environment, University of Marilia, Marília 17525-902, SP, Brazil; daniel.dbt@hotmail.com; 6 Texas Institute for Kidney and Endocrine Disorders, Lufkin, TX 75904, USA; kaportero@gmail.com (K.P.S.); tikedlufkin@gmail.com (L.A.S.); 7 Department of Internal Medicine, University of Texas Medical Branch, Galveston, TX 77555, USA

**Keywords:** glucagon-like peptide-1, non-alcoholic fatty liver disease, Alzheimer’s disease, depression, diabetes, obesity, visceral insulin resistance adiposity syndrome, Parkinson’s disease

## Abstract

Glucagon-like peptide-1 (GLP-1) is a human incretin hormone derived from the proglucagon molecule. GLP-1 receptor agonists are frequently used to treat type 2 diabetes mellitus and obesity. However, the hormone affects the liver, pancreas, brain, fat cells, heart, and gastrointestinal tract. The objective of this study was to perform a systematic review on the use of GLP-1 other than in treating diabetes. PubMed, Cochrane, and Embase were searched, and the PRISMA guidelines were followed. Nineteen clinical studies were selected. The results showed that GLP-1 agonists can benefit defined off-medication motor scores in Parkinson’s Disease and improve emotional well-being. In Alzheimer’s disease, GLP-1 analogs can improve the brain’s glucose metabolism by improving glucose transport across the blood–brain barrier. In depression, the analogs can improve quality of life and depression scales. GLP-1 analogs can also have a role in treating chemical dependency, inhibiting dopaminergic release in the brain’s reward centers, decreasing withdrawal effects and relapses. These medications can also improve lipotoxicity by reducing visceral adiposity and decreasing liver fat deposition, reducing insulin resistance and the development of non-alcoholic fatty liver diseases. The adverse effects are primarily gastrointestinal. Therefore, GLP-1 analogs can benefit other conditions besides traditional diabetes and obesity uses.

## 1. Introduction

Glucagon-like peptide-1 (GLP-1) is an incretin secreted by the distal intestinal ileum and colon L-cells following food intake. The synthesis of this molecule occurs through the proteolytic cleavage of proglucagon molecules performed by many prohormone convertase enzymes. The cleavage of proglucagon leads to the creation of two active peptides 30 or 31 amino acids long: GLP-1(7-36)-NH2 (the most commonly produced molecule) and GLP-1 (7-37) (the second most commonly produced molecule) [1].

GLP-1 has a short half-life (around 2 min), due to the action of the enzyme dipeptidyl-peptidase-4 (DPP-4), a serine aminopeptidase, and because of renal clearance, due to its low molecular weight. The DPP-4 endopeptidase is present in the membrane of cells of multiple organs (kidney, liver, pancreas, blood vessels, gut, and brain) and in a soluble form [1,2].

The biological effects of GLP-1 are mediated via binding to G protein-coupled transmembrane receptors (GPCRs) of the class B family. After hormone–receptor ligation, the levels of cAMP increase by the increased activity of adenylate cyclase, and the different actions of GLP-1 occur [1,2].

**Figure 1:**
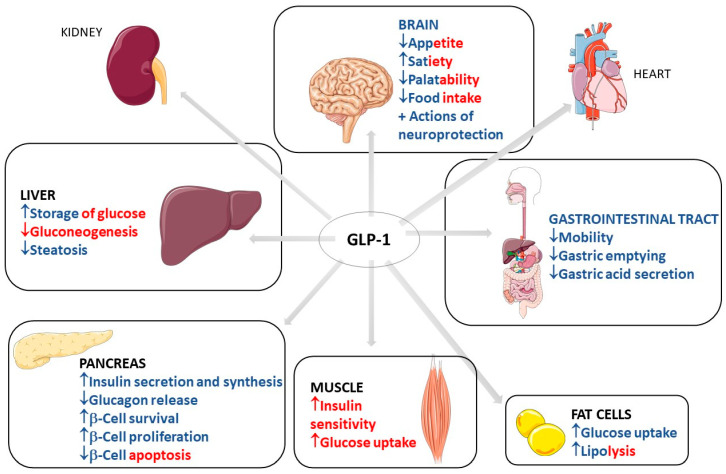
Most important traditional organ targets for GLP-1 and its actions on each target. GLP-1: Glucagon-like peptide; ↓: decrease; ↑: increase; +: plus. The red color represents the indirect effects of GLP-1 on the determined organ, and the blue color represents direct effects. The red–blue mixtures represent the determining effects in direct and indirect related GLP-1 activity.

GLP-1 has biological effects that are well known and well established in clinical medicine. These effects occur through the binding to GLP-1 receptors in multiple organs and body systems. GLP-1 action in the pancreas includes an increase in insulin secretion and synthesis, an increase in pancreatic β-cell proliferation and β-cell survival, and a decrease in glucagon release. This scenario leads to reduced gluconeogenesis and increased hepatic storage of glucose as glycogen. Other actions include increased glucose uptake by muscle cells and increased glucose uptake and increased lipolysis in adipocytes. GLP-1 can also reduce appetite, gastrointestinal motility, and gastric acid secretion. The actions of GLP-1 in the kidney include mild natriuresis. GLP-1 increases contractility and heart rate in the heart and has vascular protective effects. Due to these metabolic effects of GLP-1, it is primarily used in clinical practice to treat type 2 diabetes mellitus (T2DM) and obesity [1,2,3,4,5,6,7]. Figure 1 shows the most important traditional organ targets for GLP-1 activities and its actions on each target, with direct and indirect effects differentiated by color.

Recent clinical trials have investigated GLP-1 use beyond the traditional uses. The hormone has been used to treat depression, psoriasis, Alzheimer’s, Parkinson’s, non-alcoholic fatty liver disease (NAFLD) and non-alcoholic steatohepatitis (NASH), alcohol abuse, and its effect on adipose tissue. GLP-1rA liraglutide therapy can reduce psoriasis with concomitant type 2 diabetes severity, but may be independent of changes in weight and glycaemic control. GLP1′s effects on endothelial and adipose tissue leading to cardiovascular and neuroprotective effects are areas of particular interest. From this perspective, many new randomized clinical trials to evaluate the possible effects of GLP-1 in these areas might be undertaken in the coming years. For these reasons, this study aims to perform a systematic review of the effects of GLP1 beyond its traditional use for diabetes and body weight control [1,2,8,9,10,11,12,13,14,15,16,17,18,19,20].

## 2. Clinical Trials That Investigated the Use of GLP-1

**Figure 2:**
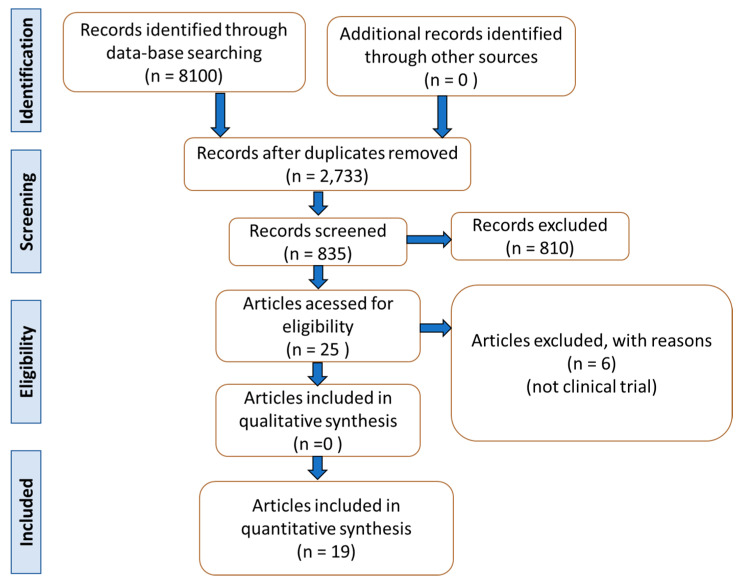
Flow chart showing the study selection.

The selection of the studies can be found in Figure 2 and Table 1. Table 2 shows the bias of each included RCT. From the 19 studies selected for the production of this article, the data of more than 1950 patients were included. Two studies concerned Parkinson’s disease [9,21], ten were related to NAFLD and NASH [18,22,23,24,25,26,27,28,29,30], four included Alzheimer’s disease patients [17,31,32,33], and three considered patients with depression [34,35,36].

Of the nineteen articles included in this review, seven were from England or the United Kingdom [9,21,22,23,34,35,36], one was from Japan [24], two were from Singapore [26,27], one was from the Netherlands [29], one was from France [28], two were from China [25,30], two were from the United States [17,33], one was multicenter [18], and two were from Denmark [31,32]. Eleven of the nineteen studies included in this systematic review had a randomized, double-blind placebo-controlled study design [9,17,18,21,22,23,29,30,31,32,33], one study had a single-center open-label comparative study design [24], one study had a prospective design [26], one study had a prospective single-center parallel-group design [28], one study had a prospective randomized study design [27], one study had a prospective randomized placebo-controlled study design [25], one study had an interventional case–control study design [35], one study was a prospective analysis [36], and one study had a matched group design [34]. Five of the nineteen studies used exenatide [9,17,21,34,36] as the GLP-1 receptor agonist, one study used semaglutide [18], and thirteen studies used liraglutide [22,23,24,25,26,27,28,29,30,31,32,33,35]. The administered doses of the GLP-1 receptor agonists ranged from microgram administrations per day to milligram administrations per day, depending on the medication (exenatide, semaglutide, or liraglutide), and the intervention period ranged from 6 weeks to 72 weeks. Some studies used an increasing dose of the GLP-1 receptor agonist until the final dose was reached.

This systematic review found that the studies included in the intervention’s comparisons showed positive effects on treating motor-defined symptoms of Parkinson’s disease, in addition to treating the non-motor symptoms of Parkinson’s. GLP-1 receptors agonists can also be associated with ameliorations in NAFLD and NASH diseases, principally due to the effects of the GLP-1 medications in resolving the histological parameters of the disease in the hepatic tissue. The use of GLP-1 analogs was associated with improvement in the metabolic parameters of insulin resistance and lipotoxicity related to the pathogenesis of NASH. The use of GLP-1 receptor agonists was also associated with reductions in visceral adiposity and attenuation of fat deposition in the liver. The use of GLP-a was associated with decreased liver cell apoptosis levels. GLP-1 receptor agonists improved impaired glucose transport across the blood–brain barrier. GLP-1 was associated with reduction of depressive symptoms and improvement in quality of life. The most common adverse effects were nausea, vomiting, and diarrhea.

## 3. Use of GLP1

### 3.1. Common Use of GLP-1: Diabetes and Obesity

The relation between obesity and DM is already well explained, and it is known that diabetes is closely related to increased cardiovascular risk. Obesity causes health problems proportionally with the increase of the individual’s body mass index (BMI), leading to increased mortality and morbidity, mainly in cases with a BMI > 30 kg/m^2^. In addition, obesity also can lead to many other outcomes, such as hypertension and abnormal lipid metabolism that leads to dyslipidemia, endothelial dysfunction, and augmented proinflammatory state [37,38].

The insulin resistance environment is produced when an abnormal ectopic deposition of fat is observed mainly in the skeletal muscles and the liver. Furthermore, genetic factors and phenotypical interactions can increase metabolic diabetes risk and contribute to the residual β-cells that cannot adequately control insulin secretion in the right proportions to overcome the insulin resistance state [37,39,40].

The usual actions of GLP-1 are summarized as controlling energy homeostasis and feeding behavior. GLP-1 regulates energy homeostasis principally by its effects on stimulating insulin secretion to control glycemic excursions and its actions on inhibiting glucagon secretion. Moreover, GLP-1 controls feeding behavior by affecting multiple neural circuits that correspond to appetite. In addition, it is known that GLP-1 affects gastric emptying and inhibits pancreatic β-cells apoptosis. Although the half-life of native GLP-1 human hormone is very short, its analogs are produced and synthesized to deliver long half-lives. The GLP-1 analog is considered beneficial in clinical practice, principally by being used in the treatment or the complementary therapy of T2DM. This insulinotropic and glucagonstatic molecule is well-tolerated and can be categorized into two groups according to the actions it promotes: short-acting compounds that deliver short-lived receptor activation and long-acting compounds that activate GLP-1 receptors continuously. GLP-1 is degraded in the peripheral parts of the human body by the dipeptidyl peptidase-4 enzyme (DPP-4). Still, the following GLP-1 analogs are produced to be resistant against the actions of this enzyme: exenatide, liraglutide, and semaglutide. Despite the use of GLP-1 DPP-4-resistant analogs in the medical routine, some drugs can suppress the actions of DPP-4, which leads to possible major actions of GLP-1. These drugs are called DPP-4 inhibitors, and the best known are sitagliptin and vildagliptin [1,38,41,42,43].

**Figure 3:**
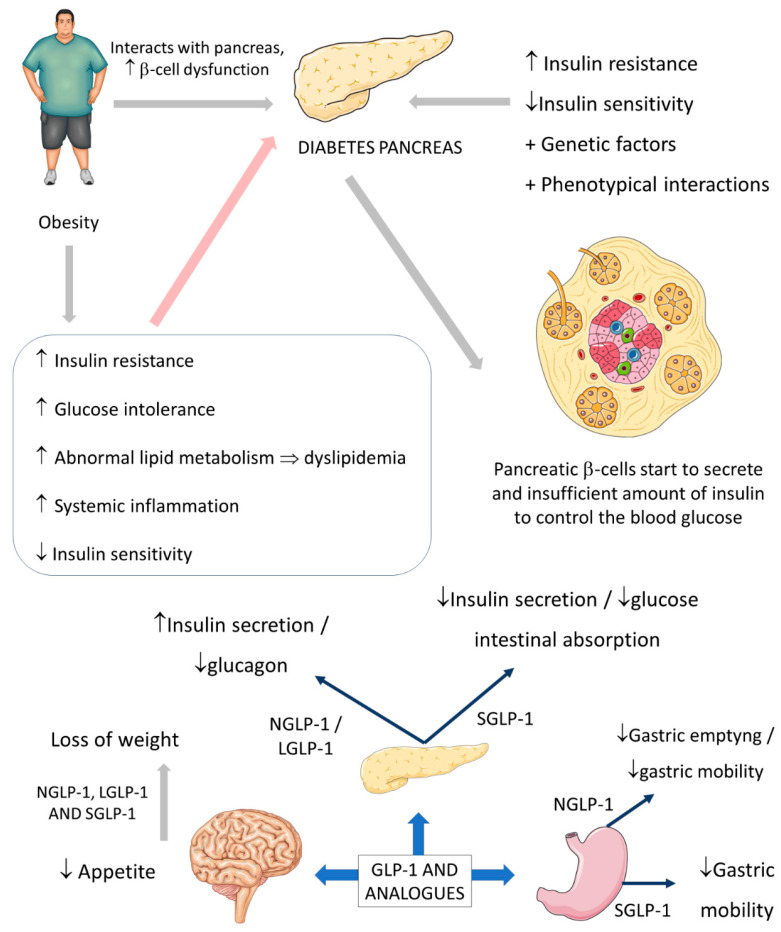
Diabetes, obesity, and possible actions of GLP-1. GLP-1: glucagon-like peptide; NGLP-1: native GLP-1; LGLP-1: long-acting GLP-1 receptor agonist; SGLP-1: short-acting GLP-1 receptor agonist; ↓: decrease; ↑: increase; ⇒: equal; +: plus.

The short-acting (2–5 h half-life) and the long-acting (several to 12 h half-life) GLP-1 receptor agonists have considerable effects on the treatment of not only diabetes but also obesity. The effectiveness of these drugs on diabetes and obesity is primarily due to the regulation of glycemia, insulin, and glucagon secretion. Short-acting GLP-1 receptor agonists have demonstrated modest reductions of fasting blood glucose levels and substantial reductions of postprandial hyperglycemia by stimulating fasting insulin secretions and decreasing glucagon secretion. In addition, these molecules reduce the gastric emptying rate and reduce appetite, leading to a reduction of the body weight by 1–5 kg on average. On the other hand, long-acting GLP-1 agonists have shown substantial decreases in fasting blood glucose levels and modest reductions of postprandial hyperglycemia with strong stimulations of fasting insulin secretions and reductions of glucagon secretion. As a result, the long-acting GLP-1 agonists may cause a decrease of body weight by 2–5 kg on average. It is known that the actions of the native human GLP-1 are different in reducing food intake and in controlling blood glucose levels in comparison to GLP-1 receptor agonists. Recent findings demonstrated that in overweight or obese adults, GLP-1 receptor agonists and phentermine–topiramate are the best drugs to reduce body weight; of the GLP-1 agonists, semaglutide might be the most effective [43,44,45,46,47]. Figure 3 summarizes the GLP-1 actions.

Besides affecting glycemia, there are other actions of the different GLP-1 and GLP-1 analogs. When the intestinal L-cells secrete GLP-1 following food intake, the incretin hormone molecules go directly to the pancreas, acting on the pancreatic β-cells leading to stimulations to increase the insulin secretion. In addition, the native molecules act directly on the pancreas to reduce the secretion of glucagon molecules. The actions of the long-acting GLP-1 receptor agonists are identical to the GLP-1 native molecules in controlling the pancreatic secretion of hormones. The native GLP-1 and GLP-1 agonists reduce bodyweight mainly by acting on the central nervous system, causing a reduction of appetite by affecting different neural mechanisms. In turn, the main actions of the short-acting GLP-1 agonists in controlling blood glucose are related to the inhibition of gastric mobility leading to delayed intestinal glucose absorption and, consequently, to a reduction of postprandial insulin secretion [1,41,43,45,46]. Moreover, changes in gut hormones, including increases in GLP-1, PYY, and oxyntomodulin, and decreases in GIP and ghrelin, or the combined actions of all these hormones, might have a role in the induction and long-term maintenance of weight loss [1,41,43,45,46,48].

### 3.2. GLP-1 and Parkinson’s Disease

**Figure 4:**
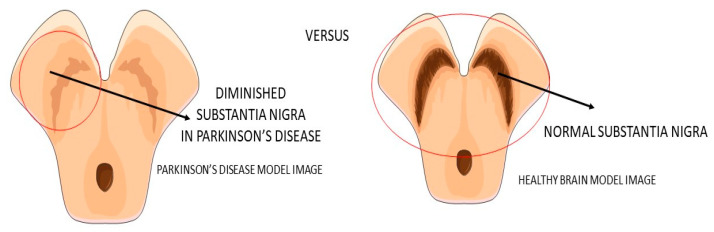
Substantia nigra in Parkinson’s disease and the healthy brain.

Parkinson’s disease (PA) is a progressive neurodegenerative disease (Figure 4) characterized predominantly by motor signals, such as tremor, akinesia, and bradykinesia. However, nonmotor symptoms and signs can also be found, such as cognitive decline, depression, signals of anxiety, sleep disturbances, and dysautonomia. It is a common neurologic ailment that affects 1.5% of individuals over 65 years old worldwide. In Parkinson’s, midbrain neurons are degenrated mainly because of mitochondrial dysfunctions that lead to cellular energy failure and, consequently, to a reduction of dopamine transmission and deposition of α-synuclein, in addition to the presence of increased oxidative stress. The deposition of a-synuclein, accumulation of reactive oxygen species (ROS), and the presence of dying neuron cells trigger an inflammatory environment in the midbrain tissue. The process leads to degeneration of the brain, together with autophagy and apoptosis. The gold standard treatments are dopamine replacement with dopaminergic medications or brain stimulation. These treatments improve the motor signals, but they are only partially effective due to the progressive nature of Parkinson’s. Subsequently, the non-dopaminergic systems will be involved in the progression of the disease, causing some nonmotor symptoms, for instance, depression and cognitive decline [49,50,51].

The two articles that focused on Parkinson’s disease were derived from the same study, which means that these two papers count only once in the final count for the total number of participants included in this present systematic review. In addition, there were correlations between the same authors in different studies referred to in this paper, principally due to the presence of the same authors in more than one study included in this review. The participants that were included in the two papers on Parkinson’s disease had idiopathic Parkinson’s [9,21]

The beneficial actions of GLP-1 go far beyond insulin secretion and appetite and include cardiovascular benefits and possibly also beneficial effects in neurodegenerative diseases. GLP-1 presents neurotrophic and neuroprotective effects demonstrated by some clinical trials studies and mouse model studies. It can cross the blood–brain barrier and central nervous system and may influence some neural actions in cellular pathways, such as neuroinflammation, mitochondrial dysfunction, and cellular proliferation. For example, the decontrolled mitochondrial functions may lead to cellular energy failure. This procedure is implicated as the leading cause of dopaminergic neuron death in Parkinson’s disease and other age-related neural dysfunction. Studies in animal models suggested that activating the GLP-1 receptors (GLP-1Rs) in the brain improves neuroinflammation, neurogenesis, and synaptic plasticity. The neurotrophic and neuroprotection promoted by GLP-1 might explain the improvements in the neuronal activities and the interference in neurodegenerative pathways, which means that GLP-1 could protect against diseases such as Alzheimer’s and Parkinson’s [49,52,53].

**Figure 5:**
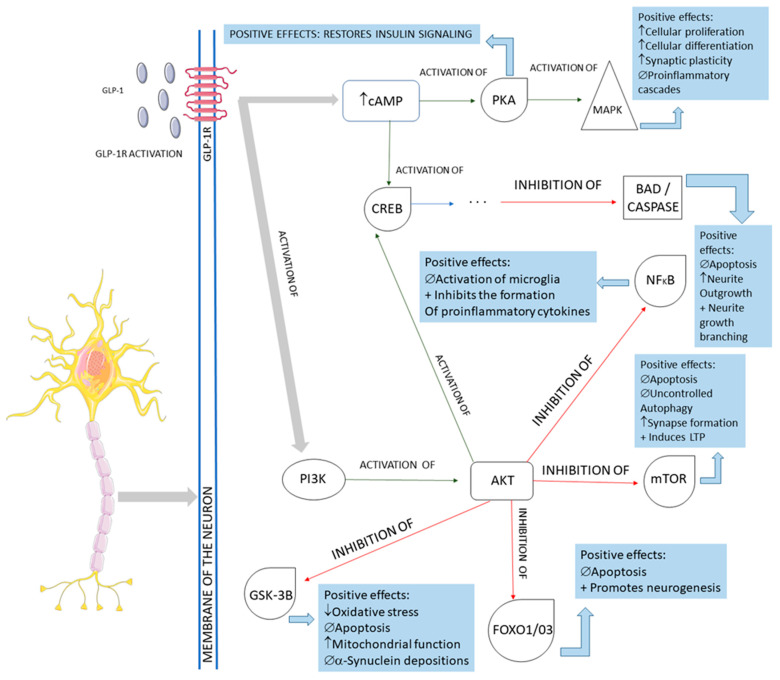
Main cellular pathways affected by GLP-1 in neurons. GLP-1R: GLP-1 receptor; cAMP: cyclic adenosine monophosphate; PKA: protein kinase A; MAPK: mitogen-associated protein kinase; CREB: cyclic adenosine monophosphate response element-binding protein; BAD: Bcl-2 antagonist of death; NFkB: nuclear factor-kappa B; PI3K: phosphoinositide 3-kinase; AKT: protein kinase B; mTOR: mammalian target of rapamycin; GSK-3B: glycogen synthase kinase 3 beta; FOXO1/03: forkhead box protein O1; ↓: decrease; ↑: increase; ∅ = impairment; +: plus.

The activation of the neurons of the central nervous system by GLP-1 and the interferences of this molecule in the pathological pathways of Parkinson’s disease are shown in Figure 5. In summary, GLP-1 molecules will bind to the GLP-1Rs located on the membrane of the midbrain’s dopaminergic neurons and activate these cells against the typical pathological pathways of Parkinson’s disease. In Parkinson’s, these midbrain neurons are degenerated mainly because of mitochondrial dysfunctions that lead to a reduction of dopamine transmission and the deposition of a-synuclein, in addition to the presence of increased oxidative stress. The deposition of a-synuclein, the accumulation of reactive oxygen species (ROS), and the presence of dying neuron cells trigger an inflammatory environment in the midbrain tissue. The GLP-1 binding on the GLP-1Rs starts several signalizing pathways in the neurons, reducing inflammation, oxidative stress, apoptosis, and a-synuclein deposition. In addition, cell proliferation restores insulin signaling and improves neuronal functions. Restoring insulin signaling in these neurons corresponds to a neuroprotective action of GLP-1 in diabetic individual neurodegeneration since T2DM subjects present insulin resistance of the brain cells that may accelerate the progression of Parkinson’s disease [9,21,49,52,54,55,56,57,58].

There is a lack of concluded clinical trials in the literature about the specific use of GLP-1 and its analogs in PA therapeutics. The study of Atahuda et al. [9] was a randomized, double-blind, and placebo-controlled trial that investigated the effects of exenatide on Parkinson’s disease. The GLP-1 analog showed positive effects on motor scores during the administration, and its benefits were sustained beyond the period of exposure. Therefore, GLP-1 agonists can modulate PA symptoms, but the mechanism involved in this process is still unknown. For these reasons, longer-term trials are necessary to clarify this relation with a more uniform and bigger sample. In a post hoc analysis, Athauda et al. [21] noted that at the end of the 48-week intervention, the participants in the exenatide group showed several improvements in domains classified by mood/depression and mood/apathy, including the domain of “emotional well-being”. Although these effects were magnified in the final stage of the intervention, these effects were not sustained after the 12 weeks of exenatide washout.

Although there are few human studies on Parkinson’s disease, numerous studies show the effects of GLP-1 in animal models [9,58,59,60,61,62]. The rat model studies demonstrated positive and effective actions of GLP-1 molecules in the rat PA models, such as neurotrophic, neuroprotective, and anti-inflammatory effects on mitochondrial gene expression and neurogenesis, improvement in synaptic functions, reduction of neuron cells apoptosis, and decreased a-synuclein neuron deposition [9,49,52,54,55,57,58,59,60,61,62,63].

### 3.3. GLP-1, Non-Alcoholic Fatty Liver Disease, and Non-Alcoholic Steatohepatitis

**Figure 6:**
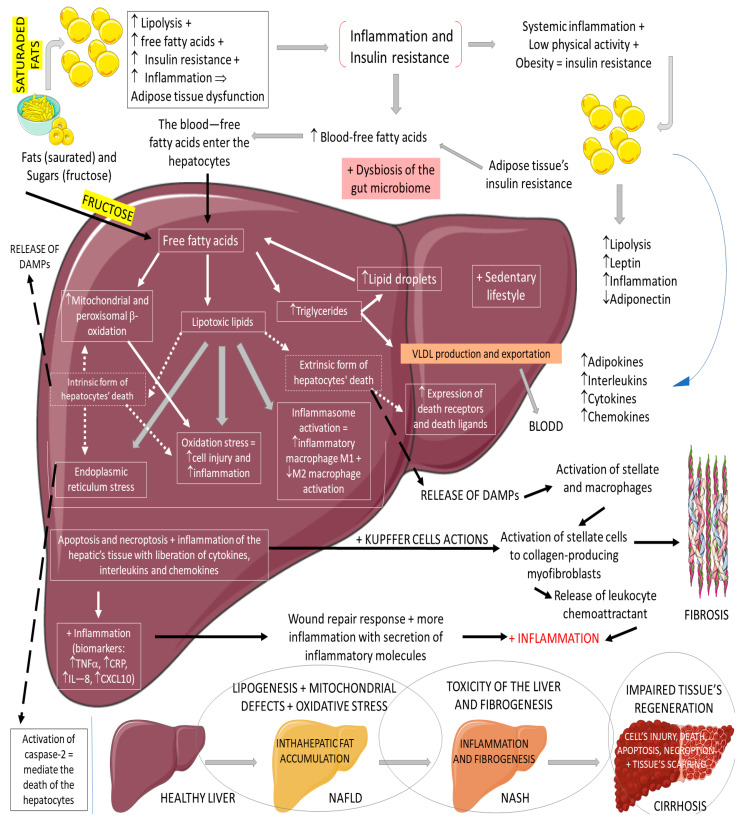
Physiopathology of NAFLD and the progression to NASH. ↓: decrease; ↑: increase; +: added to; ⇒: equal; TNF-α: tumor factor necrosis; CRP: C reactive protein; IL-8: interleukin 8; CXCL10: C–V–C motif chemokine ligand 10.

Non-alcoholic fatty liver disease (NAFLD) is the most common cause of chronic liver disease in western countries. It can be characterized by an excessive deposition and accumulation of fatty molecules in the hepatocytes in the absence of secondary causes. This accumulation can be an isolated event or accompanied by liver inflammation, which causes non-alcoholic steatohepatitis (NASH). Hepatic tissue inflammation causes progressive cell injury on the hepatocytes that can lead to fibrosis. In a variable course, the progression of the disease can lead, in addition to cirrhosis, to hepatocellular carcinoma. It is known that the massive presence of fatty molecules in the liver’s tissue is highly associated with risk factors for metabolic syndrome (MS), obesity, and insulin resistance. Although NAFLD and NASH share similar pathological pathways with alcohol-related fatty liver disease (ALD), NAFLD and NASH are considered metabolic disorders mainly characterized by the accumulation of triglycerides in hepatocytes. Nowadays, the prevalence of NAFLD and NASH is rising primarily due to the increased incidence of diabetes and MS. It is known that MS corresponds to a very high risk factor for NAFLD and NASH. MS is characterized by central abdominal obesity, systemic hypertension, insulin resistance (or T2DM), and atherogenic dyslipidemia, contributing to a prothrombotic and proinflammatory state. This scenario leads to an increase in oxidative stress (OS), which is related to mitochondrial dysfunction, accumulations of protein and oxidated lipids, and an impairment of the antioxidant system [64,65,66,67,68,69,70]. The impaired lipid metabolism in NAFLD and NASH is worsened by the increase of free fatty acid influxes to the hepatocytes and hepatic insulin resistance, in addition to a sedentary lifestyle [64,65,66,67,68,69,70]. Figure 6 summarizes the physiopathology of NAFLD and the progression to NASH.

The pharmacological therapies commonly used to treat NASH lie in no specific pharmaceuticals, such as Vitamin E and pioglitazone. It is known that vitamin E can have effects on preventing NAFLD and NASH disease progression to liver decompensation in patients with extensive fibrosis, protecting against the necessity of liver transplantation. According to Sheka et al. [71], there are many research targets for effective pharmacological therapy against NASH. GLP-1 has pleiotropic effects and can reduce liver steatosis, ameliorating NAFLD and NASH. It can have a role in the liver, adipose, and muscular tissues and the nervous system’s actions. According to Bifari et al. [64], GLP-1 analogs can limit the progression of NAFLD and NASH [64,71,72].

Since insulin resistance plays a crucial role in the pathogenesis of NAFLD and NASH, GLP-1 and its analogs have been considered a promising therapy for these conditions. They can increase hepatic insulin sensitivity (i.e., increase hepatocytes’ insulin response) by decreasing steatosis and improving liver histology. Armstrong et al. [23] showed that the use of liraglutide improved metabolic dysfunction and also improved insulin resistance and lipotoxicity in critical metabolic organs related to NASH pathogenesis. They observed improvements in several pathways, such as liver biochemistry and markers of inflammation, hepatic and systemic insulin sensitivity, insulin sensitivity and lipolysis, hepatic de novo lipogenesis, and hepatic steatosis. It has been shown that acute use of GLP-1 (mainly exenatide) reduces hepatic glucose production in healthy individuals and decreases the de novo lipogenesis of the liver tissue, in addition to reducing free fatty acids liberation from lipolysis and lowering triglyceride-derived toxic metabolites [23,64,71,73].

Furthermore, the role of GLP-1 on NAFLD might be explained by effects on body weight, inflammation and OS, and the gut–liver axis. The effects on inflammation and OS are confirmed by restoring the adipose tissue functions and ameliorating NAFLD liver abnormalities, which are added to an increase of adiponectin and a decrease of leptin levels. Adiponectin can regulate hepatic fatty acid oxidation and control enzymes, such as acetyl-CoA carboxylase and fatty acid synthase. Lastly, GLP-1 actions on the gut–liver axis can be explained by multiple pathway amelioration of postprandial lipidemia. It is known that hormonal and nutritional modulators mediate the hepatic and intestinal production of lipoproteins. GLP-1 can reduce the absorption of dietary fats by decreasing gut motility and directly inhibiting the synthesis and secretion of the chylomicron [73].

The GLP-1 actions on liver inflammation and liver fibrosis need to be further explained since NAFLD’s progression to NASH depends on the hepatic inflammatory environment. In fatty liver disease, the inflammatory environment and the inflammatory signals of inflammation are derived principally from the M1 macrophages (the proinflammatory form). It was found that GLP-1 affects the immune response and induces M2 macrophage polarization via STAT3 activation signalizing. M2 macrophages create a balance in the inflammatory liver environment by producing anti-inflammatory biomarkers. Additionally, GLP-1 increases the production of M2 macrophage-related molecules IL-10 and CD204. In addition, GLP-1 can modulate liver fibrosis and reduce the progression of NAFLD to NASH [64,73,74].

Liver fibrosis is determined by a proliferation of liver stroma cells and by alterations of the liver vasculature. Liraglutide improves the stellate cell phenotype in chronic liver disease models and promotes the de-activation of cirrhotic primary human hepatic stellate cells [64,74].

In the studies of NAFLD and NASH, the participants had NAFLD or NASH usually accompanied by obesity or T2DM, depending on the survey [18,22,23,24,25,26,27,28,29,30].

Armstrong et al. [23] found that using liraglutide for a 12-week period can be associated with amelioration of metabolic dysfunctions, insulin resistance, and lipotoxicity in key organs related to NASH. Patients underwent paired hyperinsulinemic euglycemic clamps, stable isotope tracers, adipose micro-dialysis, and serum adipocytokine/metabolic profiling. In vitro isotope experiments on lipid flux were performed on primary human hepatocytes. Liraglutide reduced body mass index, glycated hemoglobin, ALT, serum leptin, adiponectin, and CCL-2 (monocyte chemoattractant protein-1, also known as MCP-1). The use of liraglutide also increased adipose tissue insulin sensitivity, enhancing the ability of insulin to suppress lipolysis. This study showed that liraglutide might offer the potential for a disease-modifying intervention in NASH. However, the small number of participants represented the main limitation of this study.

In another study, Armstrong et al. [22] associated the use of liraglutide for 48 weeks with the histological resolution of NASH conditions, warranting extensive long-term studies. The preliminary results showed that NASH histological parameters in the liver improved in the participants treated with liraglutide. The study had a reasonable retention rate, although there were gastrointestinal disorders characterized by diarrhea, constipation, and loss of appetite. Additionally, as a strength, liver biopsies were centrally assessed by two independent and blinded pathologists.

Bouchi et al. [24], in a single-center, randomized, open-label comparative study with T2DM participants, found that the use of liraglutide for an interventional period of 36 weeks could reduce visceral adiposity, attenuating fat depositions in the liver. The reduced number of the sample represents a significant limitation in this study.

Khoo et al. [26] showed in a prospective study with NAFLD and NASH participants that the use of liraglutide for 26 weeks can lead to reductions in liver fat fractions and liver stiffness, following decreased serum levels of C reactive protein.

Smits et al. [29] showed in a clinical trial with overweight individuals with T2DM that 12 weeks of treatment with liraglutide did not reduce liver steatosis. These participants were randomized into three groups: liraglutide, sitagliptin, and placebo. Treatment with liraglutide and sitagliptin did not reduce hepatic steatosis or fibrosis in T2DM patients. Nevertheless, liraglutide reduced liver fat content, possibly due to the weight loss it promoted. The limitations of the study were untriggered magnetic resonance spectroscopy measurements and manual VOI positioning.

Petit et al. [28], in a prospective single-center study with participants affected by uncontrolled T2DM observed that six months of treatment with liraglutide reduced the liver fat content of the participants. This result was principally related to weight loss. This was not a randomized clinical trial but had a parallel-group design. Participants were assigned a 1.2 mg/day dose of liraglutide, starting at 0.6 mg/day and reaching 1.2 mg/day one week after the beginning of the study drug treatment. The authors did not evaluate liver fibrosis in the participants.

In another study by Khoo et al. [27], the participants were randomized into a supervised program of diet plus exercise to induce ≥5% of weight loss or to a liraglutide (3 mg daily) group for 26 weeks, the dose starting at 0.6 mg, reaching 3 mg at the end of 4 weeks. The obese participants in the liraglutide group showed decreased body weight, hepatic steatosis, and hepatocellular apoptosis. The intrahepatocyte aminotransferase enzyme levels were also reduced in the liraglutide group. This study did not have a control group and was open-label, which could have influenced the study’s results.

Guo et al. [25] evaluated the use of liraglutide associated with metformin for 26 weeks in subjects with T2DM and NASH. All participants (body mass index greater than 25 kg/m2) had uncontrolled diabetes and were treated with metformin as monotherapy. The study participants were randomized into insulin glargine, liraglutide, and placebo. The results showed that the intervention with liraglutide was more effective in reducing the intrahepatic content of lipids subcutaneous adipose tissue and visceral adipose tissue among the participants. Additionally, the single-center characterization of this trial can be considered a limitation of this study, which decreases its generalizability.

Yan et al. [30] investigated the effects of liraglutide, sitagliptin, and insulin glargine added to metformin on body weight and intrahepatic lipid content in subjects with T2DM and NAFLD. In combination with metformin, liraglutide reduced body weight, intrahepatic lipid content, and visceral adipose tissue. Although this study was randomized, there was a lack of a control group characterized by a placebo.

Newsome et al. [18] conducted a double-blind phase 2 trial involving participants with biopsy-confirmed NASH (and liver fibrosis at stage F1, F2, or F3) (treatment for 72 weeks). Patients were to receive once-daily subcutaneous semaglutide at a dose of 0.1 (n = 80), 0.2 (n = 78), or 0.4 (n = 82) mg or a corresponding placebo. The percentage of participants in whom the resolution of NASH was reached was 40% (0.1 mg group), 36% (0.2 mg group), 59% (0.4 mg group), and 17% in the placebo. It was seen that semaglutide treatment significantly improved the percentage of subjects with NASH resolution. No differences in the percentage of patients with improved fibrosis stage were observed.

Several studies did not use a histological biopsy to identify the intrahepatic accumulation of lipids in the participants. It is known that a liver biopsy is the gold-standard method to evaluate modifications in the lipid content in hepatocytes. This was the main limitation of the trials included in this review.

### 3.4. GLP-1 and Alzheimer’s Disease

**Figure 7:**
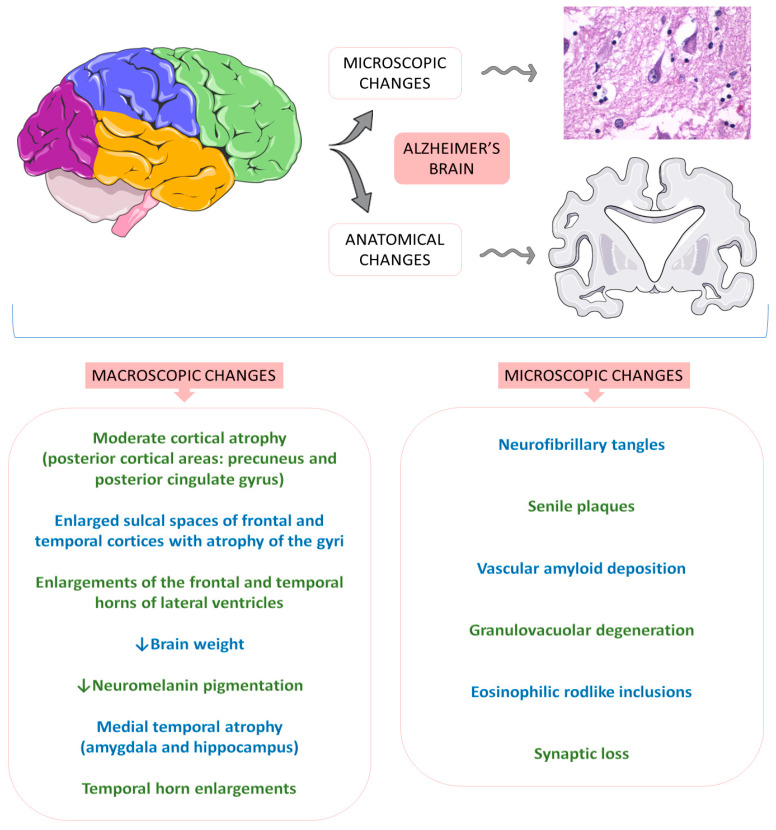
Anatomical and histological alterations in Alzheimer’s disease. ↓ = decrease.

Alzheimer’s disease (AD) is the most common neurodegenerative cause of dementia worldwide, affecting more than 10% of people over 65. AD dementia is a public health priority of the World Health Organization (WHO), probably due to its particular forms of onset and the progressive loss of cognitive and functional actions of the brain associated with age. Specifically, the first stages of AD are related to deficits in the ability to encode and store new memories. In summary, AD is characterized by cognitive insufficiencies and behavioral changes, impaired memory and learning abilities, daily functioning routines, and quality of life. Due to the lack of effective treatments for this disease, the development of a compound that interferes in a probably central pathological feature of AD is critical. Metabolic, mitochondrial, inflammatory, oxidative, and vascular changes are associated with the development of AD. However, the most significant hypothesis for the pathophysiology derives from the amyloid cascade. The amyloid precursor protein (APP) is a type I transmembrane protein synthesized primarily in the endoplasmic reticulum and posteriorly transported to the Golgi apparatus to be stored at a steady state in its final synthesis process. Subsequently, the APP molecules can be processed and cleaved at different sites by the actions of other proteases. In this protein processing, changes in amyloid cleavage can lead to the formation of an APP fragment called β-amyloid (Ab). The presence of these Ab molecules can make Ab encounter hyperphosphorylated tau protein aggregations. Consequently, this coalescence leads to a reduction in synaptic strength, loss of synaptic connections, and neurodegeneration. In addition to this interaction of proteins, it is known that AD is closely banded with peripheral and brain insulin resistance, insofar as the impaired insulin signaling in the brain contributes to amplifying neuronal dysfunctions and neurodegeneration [75,76,77,78,79]. Figure 7 shows some anatomical and histological alterations in Alzheimer’s disease.

GLP-1 is also produced in the central nervous system of humans, predominantly in the brainstem neuronal cells of the nucleus tractus solitarius of the central nervous system, to be distributed to other central nervous areas, such as the hypothalamus thalamic and cortical areas. GLP-1 triggers in vivo actions by binding to G proteins to activate cellular pathways that lead to various effects in the central nervous areas. Not only the insulin resistance of the neurons but systemic hyperinsulinemia corresponds to the harmful impact on cognitive impairment [2,75,76,77,78,79,80,81,82].

**Figure 8:**
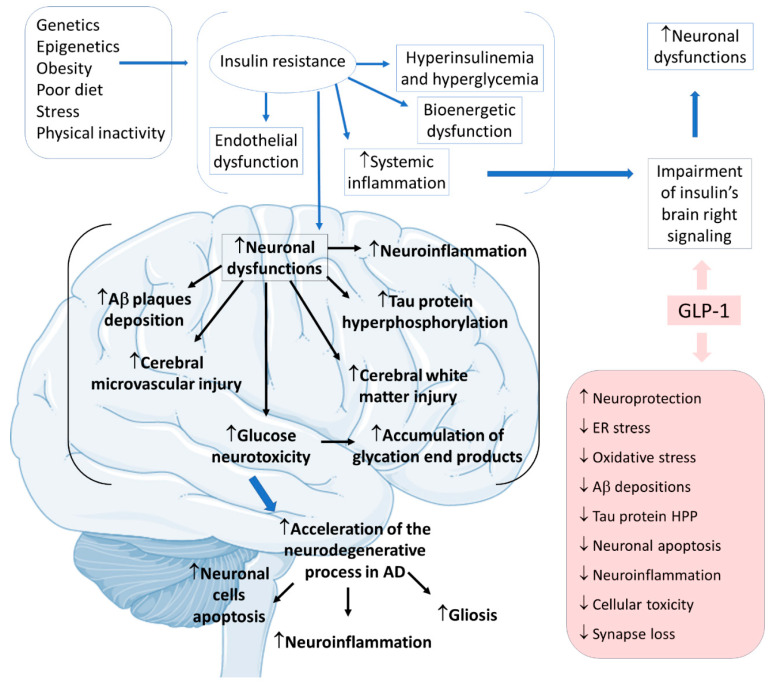
Pathophysiology of Alzheimer’s disease focused on insulin resistance and the effects of GLP-1. Ad: Alzheimer’s disease; ↓: decrease; ↑: increase; Aβ: β-amyloid; ER: endoplasmic reticulum; HPP: hyperphosphorylation.

Considering insulin resistance as a critical part of the AD pathogenic pathway, GLP-1 and its analogs can have beneficial actions on treating neurodegenerative states. GLP-1 has central effects on neuroprotection, which can be summarized by increases in neurogenesis and synaptic plasticity and decreases in neuroinflammation and protein aggregations. The pathological cascade of brain insulin resistance lies in the fact that this resistance increases Aβ plaque deposition and tau protein hyperphosphorylation in the interior of the affected AD brain areas. For these reasons, it is believed that in AD, GLP-1 reduces neuronal apoptosis, neuroinflammation, and the typical gliosis. GLP-1 can also show beneficial effects in reducing endoplasmic reticulum stress, oxidative stress, Aβ depositions, tau protein hyperphosphorylation, cellular toxicity, and synaptic loss. Furthermore, GLP-1 can amplify insulin signaling in the brain cells, leading to increased insulin sensitivity in the neurons. In addition, the insulin resistance that strengthens Aβ depositions and tau protein hyperphosphorylation can cause microvascular injury and white matter injury in the brain. Moreover, glucose neurotoxicity and accumulation of advanced glycation end products in the brain is observed. The treatment with GLP-1 can effectively prevent these events (Figure 8) [2,75,76,77,78,79,80,81,82].

Four trials that used GLP-1 to treat AD or key AD pathophysiological features were selected according to the inclusion criteria of this systematic review. In these studies, some of the participants had a diagnosis of Alzheimer’s, others were at high risk of developing Alzheimer’s, and the rest were affected by subjective cognitive complaints, but with a mini-mental exam greater than 27 points [17,31,32,33].

Mullins et al. [17] investigated the effects of exenatide for 18 months in AD parameters. The participants had no DM and were diagnosed with a high probability of having AD. The participants of the study were divided into placebo and exenatide groups (10 mcg twice daily for 18 weeks, with an initial dose of 5 mcg twice daily). The treatment with exenatide did not produce differences compared to placebo in several cognitive parameters and magnetic resonance imaging of cortical thickness and cortical volume. Of all the initial 21 participants, 18 completed the trial.

Gejl et al. [31] performed a randomized, double-blind, placebo-controlled trial to investigate the possible actions of liraglutide in restoring impaired glucose transport across the blood–brain barrier in patients with AD. The participants were randomized into two groups: liraglutide (1.8 mg daily for six weeks with an initial dose of 0.6 mg daily until the final dose was reached in the first weeks of the study) or placebo. The adverse effects of the intervention were not reported. Liraglutide improved glucose transport across the blood–brain barrier. The small number of participants could have interfered with the results. Furthermore, the post hoc analysis of the study was not blinded.

Another study by Gejl et al. [32] demonstrated that liraglutide compared to placebo did not improve amyloid deposition or cognition in participants with AD. However, it prevented the decline of glucose metabolism. This trial presents the same population as the study described above [31]. The dose of GLP-1 receptor agonists was increased from 0.6 mg daily in the initial weeks of the study to the final 1.8 mg daily dose for 26 weeks. Although the treatment with liraglutide prevented the decline of glucose metabolism in the brain, there were no differences compared to the placebo group in amyloid deposition or cognition parameters.

A randomized, double-blind, placebo-controlled study performed by Watson et al. [33] with 43 male and female subjects (45–75 y) affected by subjective cognitive complaints, but with a mini-mental exam greater than 27 showed no cognitive differences between the placebo and intervention groups. The participants were randomized into two groups: placebo and liraglutide (1.8 mg daily for 12 weeks). No adverse effects were reported, and no differences in cognition were noted. The authors suggested that the statistical thresholds used in the analyses of the study could demonstrate false positives. Although no cognitive differences were found between the studied groups after liraglutide treatment, the GLP-1 receptor agonist could improve connectivity between bilateral hippocampal areas and the brain anterior medial frontal structures of the treated patients. These changes can be associated with neuroprotective effects against AD in individuals at higher risk of developing this disease.

### 3.5. GLP-1 and Depression

Depression is a common debilitating mental condition associated with a profound impact on the quality of life, mainly due to poor management related to under-diagnosis and under-treatment. Depression is also emerging in the globalized world as one of the most important causes of morbidity and mortality. Although depression is a heterogeneous syndrome that comprehends complex pathogenesis, some neurobiological factors influence this condition. In addition, anatomical modifications in the prefrontal cortex, amygdala, hippocampus, and all areas of the central nervous system can contribute to the development of this condition. Furthermore, it is known nowadays that genetic and environmental factors are also involved in the pathophysiology of depression. However, despite all the research in this area, this condition is still not fully understood. Recent studies have shown the relationship between depression and metabolism, since diabetes and obesity were already considered conditions related to a significant risk factor for neurophysiological conditions. In summary, depressive diseases correspond to somatic and cognitive alterations principally including sadness, empty moods, irritability, or anhedonia. It is known that if patients affected by depression do not receive adequate treatment, the depressive symptoms aggravate and serious outcomes can emerge [83,84,85,86,87].

Neuroinflammation is one condition intimately related to depression due to sustained stress. The peripheral levels of inflammatory markers can correspond to the severity of depression. Patients with depressive disorders exhibit high levels of proinflammatory cytokines, such as IL-1β, TNF-α, IL-6, and IL-1, followed by CRP elevation. In addition, neuroinflammation is associated with an increase in OS in the brain. The brain presents high amounts of polyunsaturated fatty acids in its composition, inadequate anti-oxidative machinery, and consumes large amounts of oxygen. Chronic neuroinflammation is related to serotonergic, dopaminergic, and noradrenergic dysfunctions, and the modulation of inflammatory conditions can be associated with ameliorations in depressive symptoms. GLP-1 can potentially protect against neuroinflammation principally by controlling the production of proinflammatory cytokines and reducing OS [84,85,88].

The neurotransmitter imbalance (serotonergic, dopaminergic, and noradrenergic dysfunctions) is directly related to neuroinflammation and can lead to behavioral changes and behavioral impairment. The stimulation of GLP-1 receptors in the cerebral cortex and hippocampus is related to the modulation of serotonin, dopamine, gamma-aminobutyric acid, and glutamate. Additionally, GLP-1 stimulates dopamine turnover in the amygdala due to its actions on dopamine receptor 2. Due to these effects as a neurotransmitter release supporter, GLP-1 can be related to the amelioration of depressive behavioral signals and symptoms [84,85,88].

**Figure 9:**
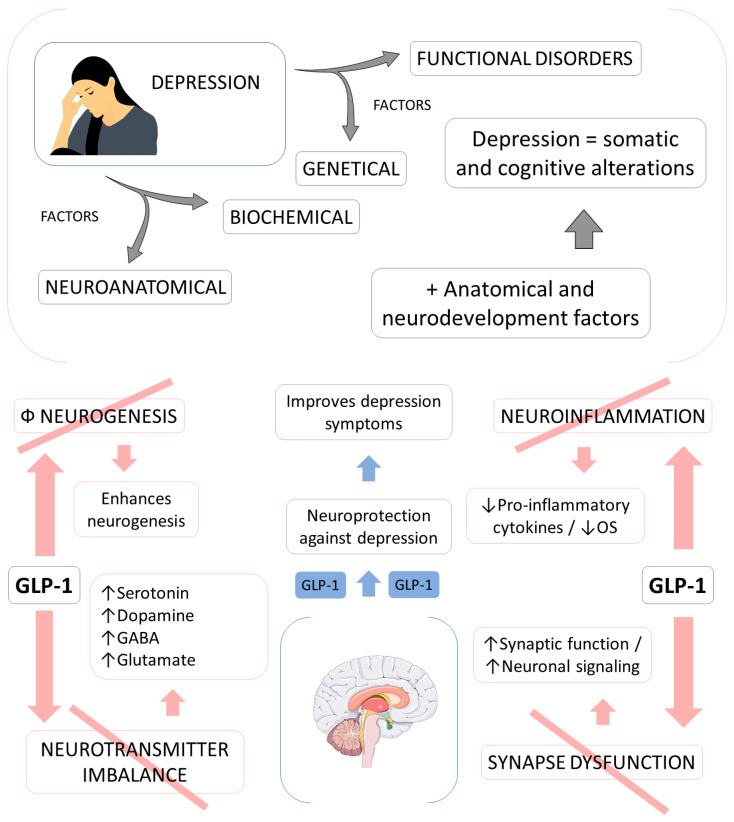
The actions of GLP-1 in key factors involved in the pathophysiology of depression. ↑ = increase; ↓ = decrease; GABA: gamma-amminobutyric acid; OS: oxidative stress; +: plus; Φ: impairment.

Depression is related to impairments due to neurogenesis, principally in the hippocampus, as well as neuronal atrophy and a decreased volume of several neuronal areas, such as the hippocampus, cortex, and limbic lobe regions of the brain. These regions are related to emotion and cognition in the normal brain. Decreased hippocampus volume and hippocampus atrophy are related to depressive episodes in patients affected by depression. Past studies demonstrated that the stimulation of neurogenesis in the hippocampus influences emotional regulation and depression, acting as an anti-depressant molecule due to increased serotonin receptors’ increased activity and reduced neuronal loss by neuronal replacement. GLP-1 can stimulate neurogenesis by several different pathways. Studies with human neuronal cells could demonstrate that GLP-1 increases cell proliferation, and studies with animal models could indicate the stimulation of the neuronal cells by GLP-1 and by the activation of GLP-1 receptors. In rodent models, liraglutide promoted neurogenesis in the hippocampus. In mouse models, neurogenesis was activated by the GLP-1 stimulations of cAMP/PKA and Akt/GSK3 cellular signaling pathways [84,85,88]. Figure 9 shows some effects of GLP-1 in the pathophysiology of depression.

The depressive brain is related to the impairment of synaptic function, synaptic plasticity, and neuronal loss due to the loss of neuronal proteins. Depression is also related to synaptic dysfunctions, which lead to impairments of neural circuits due to flawed communications pathways between the neurons. It is possible that depression and the neurogenesis impairment this condition brings lead to disruptions of physiological mechanisms related to controlling synaptic function, such as synaptic plasticity and synaptic connections. GLP-1 improves memory and cognitive functions in depressive patients by stimulating synaptic function and stimulating neuronal signal transmissions [84,85,88,89]. In the included RCTs of depression, the improvements of depressive symptoms were studied in individuals affected by different diseases, such as type 2 diabetes mellitus and polycystic ovary syndrome [34,35,36].

KAHAL et al. [35] evaluated the effects of liraglutide on quality of life and depression in an interventional case–control study with 36 obese women divided into two groups: with or without polycystic ovary syndrome. All participants received 0.6 mg liraglutide once daily for one week, followed by 1.2 mg once daily for one week, followed by 1.8 mg once daily for six months. The treatment with liraglutide improved the quality of life in the participants affected by polycystic ovary disease. However, no differences were found in the risk for depression of these women.

Moulton et al. [36] discussed incretin-based therapies as a novel treatment for depression in T2DM. The study divided the participants into two groups: incretin and control. In the incretin group, the participants received the following medications: sitagliptin (n = 15), saxagliptin (n = 6), exenatide (n = 3), and vildagliptin (n = 1). The results showed that the use of the incretin-based therapy could be associated with ameliorations in the depressive symptoms of newly diagnosed T2DM patients. This study had a very small number of participants using a GLP-1 receptor agonist treatment. The control group had 837 participants. Notably, the sample sizes of the two groups were different and could have interfered with the results. Moreover, the adverse effects were not reported. In addition, the follow-up of this trial had some limitations, considering that most patients did not have a categorical definition of their depressive symptoms at the baseline of the study, and the precise duration of the antidiabetic therapies used by the participants was not reported.

Grant et al. [34] investigated the psychological and quality of life changes of the use of exenatide in a matched group design study with participants diagnosed with T2DM who did not reach adequate glycemic control levels with glucose-lowering oral therapies. The participants were divided into two groups: insulin and exenatide. This trial was not randomized. The adverse effects were not reported, and no dropouts were registered. The results showed that participants treated with GLP-1 receptor agonists experienced reductions in the depression scale used in the study compared to the insulin group.

### 3.6. GLP-1: Other Uses

The primary actions of GLP-1 and GLP-1 receptor agonists in metabolism homeostasis are mainly related to the maintenance of glucose tolerance, which shows these molecules to be extremely powerful in improving glucose regulation in individuals affected by (primarily) T2DM [45,90]. However, GLP-1 can be considered to treat conditions other than T2DM, including NAFLD, NASH, PA, AD, and depression. Iepsen et al. [91] found that liraglutide has positive effects on bone formation and prevention of bone loss. Hygum et al. [92] found that using liraglutide in T2DM patients prevented changes in bone resorptions, preserving hip bone mineral density. One double-blind, placebo-controlled study showed that GLP-1 can decrease aldosterone secretion in healthy individuals suggesting that GLP-1 can act on blood pressure and the renin–angiotensin–aldosterone system [93].

#### GLP-1 and Chemical Dependency

Alcohol addiction affects approximately four percent of the population and contributes significantly to the global burden of diseases with substantial costs to society. Addiction development is related to reward-related dopamine levels in the nervous system; thus, it can be triggered by substances and behaviors like gambling or sexual addiction. GLP-1 agonists also act at the mesolimbic system, decreasing the compulsion of alcohol consumption, being a possible adjuvant to the mental and behavioral disorders due to alcohol use with withdrawal syndrome. Nevertheless, few studies investigated the patterns in different types of addiction; most have demonstrated the action only in cocaine and alcohol abuse [94,95].

Alcohol consumption acts on the nervous system reward-related areas, more precisely at the nucleus accumbens and in the ventral tegmental area, in which it inhibits dopamine release. Several animal experiments were undertaken to investigate the effects of GLP-1 receptor agonists on many related behavioral aspects of rats under alcohol conditions. In acute treatment, the results showed that liraglutide could suppress alcohol-induced accumbal dopamine release in mice. In addition, liraglutide acutely prevented effects of alcohol deprivation and reduced alcohol intake viewed in outbred rats. Thus, GLP-1 receptor agonists could attenuate the impact of alcohol withdrawal, with a positive impact in reducing relapses—a critical obstacle to dependency treatment. Therefore, the appearance of the new drug has been attracting attention in the treatment of mental and behavioral disorders due to alcohol use with withdrawal syndrome since these disorders have a number of impacts in the biopsychosocial context [95,96,97].

Recent preclinical evidence suggests that GLP-1 receptor agonists could be repurposed to treat cocaine craving-induced relapse. Although more human trials are needed it provides important information about posology, route administration, and possible adverse effects [98]. In rodents, liraglutide reduces alcohol withdrawal symptoms, prevents acute alcohol from activating the mesolimbic dopamine system, and reduces various alcohol drinking behaviors. In addition, liraglutide decreases alcohol intake in vervet monkeys. In humans, studies are still limited [96].

**Figure 10:**
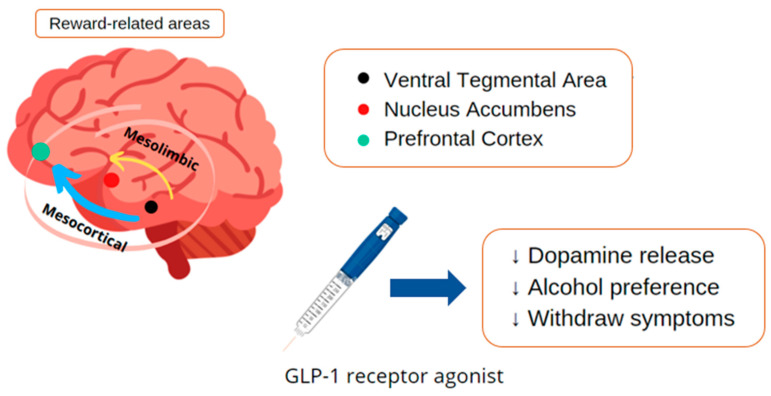
Considerations about the role of GLP-1 receptor agonists in the pathophysiology of chemical dependency. ↑: increase; ↓: decrease.

Studies that evaluate patients with addiction problems usually have many dropouts; the population is still stigmatized and mostly lives in social vulnerability, hindering studies in the area. A recent study assessed the short-term impacts of cocaine use following GLP-1 receptor agonist administration. It is difficult to follow these patients longitudinally, so this study took the first step. However, the small sample with only thirteen patients and a punctual evaluation after using the GLP-1 receptor agonist did not demonstrate significance in the self-administration of the drug and the subjective effect of the drug in patients with cocaine use disorder [99]. Figure 10 shows some considerations about the role of GLP-1 receptor agonists against chemical dependency.

## 4. Materials and Methods

### 4.1. Focal Question

The focal question that was considered in our review was, “What are the effects of GLP-1 analogs in conditions other than diabetes and obesity?”

### 4.2. Language

We included only trials published in English. Although this may be a limitation, a review pointed out there is no evidence of a systematic bias in literature reviews due to language restrictions [100].

### 4.3. Databases

We consulted PubMed, EMBASE, and COCHRANE databases for building this review. The mesh terms were GLP-1 analogs, exenatide, semaglutide, liraglutide, lixisenatide, dulaglutide, Parkinson’s disease, Alzheimer’s disease, non-alcoholic fatty liver disease, non-alcoholic steatohepatitis, and depression. The mesh terms enabled the search and identification of clinical studies that were related to the objectives of this review. PRISMA guidelines (Preferred Reporting Items for a Systematic Review and Meta-Analysis) were followed [101] (Figure 2).

### 4.4. Study Selection

For this systematic review, we included randomized clinical trials (RCTs) and non-randomized clinical trials (nRCTs) that investigated the effects of GLP-1 analogs on conditions other than the traditional use. The inclusion criteria included double-blind and placebo-controlled studies, randomized and open-label studies, prospective and parallel-group design studies, prospective-randomized and placebo-controlled studies, interventional case-control studies, and matched group design studies. Only full texts were considered.

The exclusion criteria were animal models, in vitro studies, studies published in a language other than English, reviews, poster presentations, case reports, and editorials. Descriptive and systematic reviews helped to build the discussion section.

### 4.5. Data Extraction

We did not restrict the period for searching RCTs and nRCTs. The included studies can be seen in Table 1.

### 4.6. Quality Assessment

The risk of bias evaluation was performed for each included RCT (detection, selection of the trial, and reporting of bias) according to the Cochrane Handbook for Systematic Reviews of Interventions for quality assessment [102].

## 5. General Comments

We can conclude that GLP-1 analogs play a broader role than appetite modulation for the treatment of T2DM. Considering the increase in life expectancy and the common sedentary lifestyle behavior in modern society, measures that protect against several diseases have an important impact. GLP-1 can play an important role in different metabolic disorders, such as non-alcoholic fatty liver disease and non-alcoholic steatohepatitis, decreasing visceral adiposity and fat deposition in the liver, and insulin resistance in non-alcoholic liver disease.

In addition, GLP-1 analogs lead to improvements in brain glucose metabolism and glucose transport across the blood–brain barrier in Alzheimer’s disease. They can alleviate depressive symptoms, and be used to improve quality of life and depression scales. Studies that evaluated the action of GLP-1 analogs in Parkinson’s disease showed positive results in the motor and behavioral sphere triggered by comorbidity. Recent research also indicates that GLP-1 analogs may play a role in treating chemical dependency, inhibiting dopaminergic release in the brain’s reward centers, decreasing withdrawal effects and relapses in animal models.

Our results suggest further studies, considering the broad action of GLP-1 analogs in several comorbidities. Studies in the literature addressing the unconventional use of GLP-1 analogs are still scarce and have been performed primarily on animal models. More studies in humans should better elucidate matters of dosage, possible routes of administration, and other benefits and side effects.

## Figures and Tables

**Table 1:** Effects of GLP-1 receptor agonists against different human diseases.

Reference	Local	Patients	Intervention	Outcomes	Adverse Effects	Observations
GLP-1 and Parkinson’s Disease						
Athauda et al. [9]	England	Randomized, double-blind, placebo-controlled, single-center clinical trial with 62 male and female participants (25–75 y) with idiopathic PD.	Participants were randomized into 2 groups: exenatide (32.2 mg/w) or placebo/48 weeks, followed by a 12 w washout period.	Exenatide represented positive effects on defined off-medication motor scores in PD.	Gastrointestinal symptoms (nausea, constipation, abdominal pain), injection site reaction, lower urinary symptoms, back pain, upper respiratory tract infection, loss of appetite, vomiting, dyskinesia, and anxiety in the exenatide group. Gastrointestinal symptoms and injection site reactions were common in the placebo group.	62 participants were initially randomized to the study, but 60 participants had their results on the primary analysis.
Athauda et al. [21]	England	Post hoc analysis of a randomized, double-blind, placebo-controlled study with 62 male and female patients diagnosed with idiopathic PD.	Participants were randomized into 2 groups: exenatide (2 mg, 1xd weekly for a 48 w period, followed by a washout design of a 12 w period) or placebo.	Participants of the exenatide group had better improvements in mood/depression and mood/apathy. Emotional well-being was improved in the exenatide group. However, these results were not sustained after the 12 w period of exenatide washout.	Gastrointestinal, injection site reaction, lower urinary symptoms, back pain, upper respiratory-tract infection, postural hypotension, loss of appetite, vomiting, dyskinesia, and anxiety in the exenatide group.	62 participants were initially randomized to the study, but 60 participants had their results on the primary analysis. This information was viewed in the original article of the study.
GLP-1, NAFLD, and NASH						
Newsome et al. [18]	Multicenter	Randomized, double-blind, placebo-controlled, parallel-group, multicenter clinical trial with 320 male and female subjects (18–75 y, 20–75 y in Japan) affected by histological evidence of NASH, with or without T2DM. All patients had BMI > 25.	Participants were randomized into 4 groups: semaglutide 0.1 mg 1xd/72 w followed by a 7 w follow-up) (n = 80, 55.2 ± 10.9 y, 51 female, BMI average of 36.1 ± 6.4); semaglutide 0.2 mg 1xd/72 w followed by a 7 w follow-up), semaglutide 0.4 mg 1xd/72 w followed by a 7 w follow-up), (n = 82, 54.3 ± 10.2 y, 47 female, BMI average of 35.2 ± 6.6, and placebo (n = 80, 52.4 ± 10.8 y, 44 female, BMI average of 36.1 ± 6.6), and placebo (same administration).	Semaglutide use was significantly related to the percentage of patients’ NASH resolution compared to placebo. The intervention of the study did not show results on the fibrosis stage of the participants.	Nausea, constipation, decreased appetite, abdominal pain, and vomiting in the semaglutide 0.4 mg group. Among other effects, neoplasms were reported in 15% of the semaglutide groups and 8% of the placebo participants.	The clinical trial started with 320 participants, but 302 completed the trial (94% of the initial total of subjects) and 285 participants completed the treatment (89% of the initial total of subjects). 277 subjects (87% of the initial total) had their results compounding the primary and the confirmatory secondary analysis of the outcomes. As a positive point of this study, the authors investigated the effects of different doses of semaglutide against NASH.
Armstrong et al. [23]	England	Randomized, double-blind, placebo-controlled clinical trial with 14 participants (18–70 y, BMI ≥ 25 kg/m^2^) diagnosed with NASH.	Participants were randomized into 2 groups: liraglutide (n = 7, 1.8 mg liraglutide injections 1xd/12 weeks) and placebo (n = 7). The dose started at 0.6 mg daily until reaching 1.8 mg/d a few days after the clinical trial.	Liraglutide was associated with reductions of metabolic dysfunctions, insulin resistance, and lipotoxicity in key organs related to the pathogenesis of NASH.	NR	This study presented a small number of participants.
Armstrong et al. [22]	England	Randomized, double-blind, placebo-controlled phase 2 clinical trial with 52 participants (18–70 y) affects by NASH and with BMI ≥ 25 kg/m^2^.	Participants were randomized into liraglutide (1.8 mg, n = 26) and placebo (n = 26) groups 1xd/48 weeks.	The use of liraglutide led to histological resolution of NASH.	Gastrointestinal disorders: diarrhea, constipation, and loss of appetite.	There were 3 participants in the liraglutide group who abandoned the treatment.
Bouchi et al. [24]	Japan	Single-center, randomized, open-label, comparative study with 19 patients with T2DM older than 20 years.	Participants were randomized into 2 groups: liraglutide + insulin (n = 9, liraglutide, increasing doses until 0.9 mg/d, 63% male, 57 ± 16 y) and insulin alone (n = 10, 33 male, 60 ± 22 y)/36 w; some participants had their doses adjusted between the 24th and the 36th w.	The use of liraglutide could reduce visceral adiposity and attenuate fat deposition in the liver.	No severe adverse events were observed in either group.	There were 2 patients who did not conclude the study, 1 in the liraglutide group and the other in the placebo group. This study presented a small number of participants.
Khoo et al. [26]	Singapore	Prospective study with 24 participants (21–65 y) diagnosed with NAFLD and NASH had the criteria of obesity.	Diet and exercise group (n = 12): each individual was told to reduce caloric intake and to exercise moderately over 26 weeks. Liraglutide group (n = 12): not given individual diet or exercise and advised to continue the pre-study routine/26 weeks, starting at 0.6 mg until 3 mg.	Weight, total fat mass, and insulin resistance decreased significantly in both groups. Reductions in serum ALT and AST, LFF, and liver stiffness were also significant and similar. CRP decreased only in liraglutide group.	Liraglutide group: nausea, transient abdominal discomfort, and bloating with every dose increment.	This study presented a small number of participants.
Smits et al. [29]	Netherlands	Single-center, randomized, double-blind, placebo-controlled, double-dummy, three-armed, parallel group clinical trial with 52 overweight subjects (35–75 y) with diagnosis of T2DM.	The participants were randomized into 3 groups: 1xd liraglutide (n = 17, 1.8 mg, 60.8 ± 1.8 y, 12 men), 1xd sitagliptin (n = 18, 100 mg, 61.5 ± 1.7 y, 14 men) and 1xd placebo (n = 17, 65.8 ± 1.4 y, 13 men)/12 w.	Treatment with liraglutide and sitagliptin did not reduce hepatic steatosis or fibrosis in the T2DM patients.	No serious adverse effects occurred.	51 participants completed the study (one participant of the sitagliptin group did not conclude the trial).
Petit et al. [28]	France	Prospective single-center parallel-group study with 68 patients (56.9 ± 11.3 y, 31 female) with uncontrolled T2DM. 80 participants initiated the trial.	Liraglutide 1.2 mg/day for 6 m (dose that started at 0.6 mg/d and after 1 week reached 1.2 mg/d, 37 men, 56.9 ± 11.3 y). The study intervention took 6 m to be completed.	Liraglutide reduced liver fat content. The effects may be derived from the loss of weight.	6 participants in the liraglutide group did not complete the study because of gastrointestinal adverse effects.	80 participants initiated the study, 68 participants completed the trial.
Khoo et al. [27]	Singapore	Prospective randomized study with 30 obese participants with NAFLD (40.7 ± 9.1 y), BMI 33.2 ± 3.6 kg/m^2^ (90% male).	Participants were randomized to a supervised program of diet + exercise to induce ≥5% of weight loss (n = 15) or to liraglutide 3 mg daily (n = 15, the dose started at 0.6 mg and reached 3 mg at the end of 4 w)/26 w.	Liraglutide could decrease body weight, hepatic steatosis, and hepatocellular apoptosis in obese participants with NAFLD. There were decreases in serum alanine aminotransferases and reductions in caspase cleaved cytokeratin-18.	Nausea, abdominal discomfort and bloating, diarrhea, flatulence, constipation, dizziness, and muscle aches in the liraglutide group.	3 participants did not conclude the study (1 of the liraglutide group).
Guo et al. [25]	China	Single-center, prospective, randomized, placebo-controlled stud with 96 T2DM and NASH patients (30–60 y), BMI greater than 25 kg/m2, and treated with metformin as monotherapy.	Participants were randomized into 3 groups: insulin glargine group (n = 32, 18 male, 52.0 ± 8.7 y), liraglutide (n = 32, 16 male, 53.1 ± 6.3 y) and placebo (n = 32, 20 male, 52.6 ± 3.9). The study intervention took 26 weeks to be completed.	Compared with the placebo group, treatment with liraglutide plus an adequate dose of metformin for 26 weeks is more effective in the reductions of intrahepatic content of lipids, subcutaneous adipose tissue, and visceral adipose tissue.	Nausea, vomiting, and diarrhea were gastrointestinal effects reported. Hypoglycemia was one adverse effect reported in the liraglutide group.	91 patients completed the trial: 30 in the insulin group, 31 in the liraglutide group and 30 in the placebo group.
Yan et al. [30]	China	Randomized, double-blind, placebo-controlled trial with 75 participants (30–75 y) with T2DM and affected with NAFLD. The participants had been treated with metformin monotherapy.	Participants were randomized into 3 groups: liraglutide group (n = 24, 7 female, 1.8 mg 1xd), sitagliptin (n = 27, 6 female, 100 mg 1xd), and insulin glargine (n = 24, 10 female)/26 w.	Metformin and liraglutide reduced body weight, intrahepatic lipids, and visceral adipose tissue. There was an improvement in glycemic control with the use of liraglutide.	Nausea, vomiting, and headache were reported to the use of liraglutide.	65 participants completed the trial: 18 in the liraglutide group, 26 in the sitagliptin group, and 21 in the insulin glargine group.
GLP-1 and Alzheimer’s Disease						
Mullins et al. [17]	United States	Randomized, double-blind, placebo-controlled phase 2 clinical trial with 27 male and female subjects (older than 60 years) with absence of DM diagnosed with high probability for AD.	Participants were randomized into 2 groups: placebo (n = 10, 74.0 ± 6.4 y, 4 male) and exenatide (n = 11, 71.7 ± 6.9 y, 7 male, initial dose of 5 mcg/2xd, which was augmented after 1 week of the start of the study for 10 mcg/2xd)/18 w.	Exenatide demonstrated no differences or trends compared to placebo in the parameters of comparison used by the study. The treatment with the GLP-1 receptor agonist did not produce differences in comparison with the placebo intervention in clinical and cognitive measurements, in magnetic resonance imaging cortical thickness, or in cortical volume, nor were there differences in plasma biomarkers and in plasma neuronal extracellular vesicles, except with Aβ42 present in the extracellular vesicles.	Nausea, diarrhea, abdominal pain, transient asymptomatic elevation of pancreatic enzymes, loss of appetite, loss of weight, hypoglycemia.	27 participants were randomized, but only 18 participants completed all the study.
Gejl et al. [31]	Denmark	Randomized, double-blind, placebo-controlled trial with 38 participants diagnosed with AD.	Participants were randomized into 2 groups: liraglutide (n = 18) and placebo (n = 20, 1,8 mg/d)/6 w. The dose of liraglutide was increased from 0.6 mg to 1.8 mg daily in the initial weeks.	Liraglutide treatment improved glucose transport by the blood–brain barrier.	NR	17 participants in the placebo group and 14 participants in the liraglutide group completed the study.
Gejl et al. [32]	Denmark	Randomized, double-blind, placebo-controlled clinical trial with 38 male and female participants with AD.	Participants were randomized into 2 groups: placebo (n = 20, 66.6 y, 5 female) and liraglutide 1.8 mg d (n = 18). The dose was increased from 0.6 mg to 1.8 mg in the initial weeks of the study/26 w.	No differences between the placebo and liraglutide groups with respect to amyloid depositions or cognition, but the treatment with liraglutide prevented the decline of glucose metabolism.	Liraglutide: gastrointestinal effects such as nausea, loss of weight, a decrease of systolic blood pressure, and reduction of adipose tissue.	38 participants initiated the study, 34 participants completed the trial.
Watson et al. [33]	United States	Randomized, double-blind, placebo-controlled clinical trial with 43 male and females subjects (45–75 y) affected by subjective cognitive complaints, but with a mini-mental exam greater than 27.	Participants were randomized into 2 groups: placebo (n = 21) and liraglutide (n = 22). The dose of liraglutide was increased from 0.6 mg to 1.8 mg d/12 w.	There were no cognitive differences between the groups after the 12 weeks.	NR	The first analysis of the study counted 41 participants, although the second counted 32 participants.
GLP-1 and Depression						
Kahal et al. [35]	United Kingdom	Interventional case–control study with 36 obese women with or without polycystic ovary syndrome (PCOS).	Participants had POS diagnosis (n = 19, 33.9 ± 6.7 y, 102.1 ± 17.1 kg) or not (control, n = 17, 33.5 ± 7.1 y, 100.4 ± 15.1 kg). All participants received liraglutide: 0.6 mg 1xd/1 w, followed by 1.2 mg once daily for 1 w, followed by 1.8 mg 1xd/6 m.	The treatment with liraglutide improved the quality of life in the obese participants affected by PCOS, but no differences were found for risk of depression or need for treatment.	Nausea and vomiting.	25 participants completed the trial: 13 in the PCOS group and 12 in the control group.
Moulton et al. [36]	United Kingdom	Prospective analysis of baseline and of 1-year period of data from 862 male and female participants newly diagnosed with T2DM (within 6 months prior to the study’s recruitment, 55.2 ± 10.5 y).	There were 2 groups: incretin (n = 25, 50.2 ± 8.8 y, 14 male, 15 sitagliptin, 6 saxagliptin, 3 exenatide, and 1 vidagliptin) and control (n = 837, oral hypoglycemic agents or insulin, 476 male, 55.4 ± 10.5 y)/12 m.	The use of an incretin-based therapy for newly diagnosed patients with T2DM was associated with improvements in depressive symptoms.	NR	1735 participants were eligible to be part of the study and 1444 participants completed the follow-up after 1 year. The 1444 participants who completed the 1-year follow-up were more likely to have prescribed anti-depressant medications.
Grant et al. [34]	United Kingdom	Matched group design study with 138 male and female participants diagnosed with T2DM and did not reach adequate glycemic levels with glucose-lowering oral therapy.	Participants were divided into 2 groups: insulin (n = 67, 59.12 ± 8.6 y, 33 male) and exenatide (n = 71, 57.81 ± 9.5 y, 41 male)/18 w, but the data were collected over the 6 m of study duration.	Participants treated with exenatide had significant reductions in the depression scale used in the study compared to the insulin group.	NR	No dropouts were registered during the study.

Y = years, mg = milligrams, mcg = micrograms, n = number of subjects, BMI = body mass index, NASH = non-alcoholic steatohepatitis, NAFLD = non-alcoholic fatty liver disease, kg = kilograms, ALT = alanine aminotransferase, AST = aspartate transaminase, LFF = liver fat fraction, T2DM = type 2 diabetes mellitus, PCOS = polycystic ovary syndrome, NR = not reported.

**Table 2:** Descriptive results of the biases found in the included clinical trials.

Study	Question Focus	Appropriate Randomization	Allocation Blinding	Double-Blind	Losses (<20%)	Prognostics or Demographic Characteristics	Outcomes	Intention to Treat Analysis	Sample Calculation	Adequate Follow-Up
Athauda et al. [9]	Yes	Yes	Yes	Yes	Yes	Yes	Yes	No	Yes	Yes
Athauda et al. [21]	Yes	Yes	Yes	Yes	Yes	Yes	Yes	No	Yes	Yes
Newsome et al. [18]	Yes	Yes	Yes	Yes	Yes	Yes	Yes	Yes	Nr	Yes
Armstrong et al. [23]	Yes	No	Yes	Yes	Nr	Yes	Yes	Yes	No	Yes
Armstrong et al. [22]	Yes	Yes	Yes	Yes	Yes	Yes	Yes	No	Yes	Yes
Bouchi et al. [24]	Yes	No	No	No	Yes	Yes	Yes	No	No	Yes
Khoo et al. [26]	Yes	Yes	No	No	NR	Yes	Yes	Yes	Nr	Yes
Smits et al. [29]	Yes	Yes	Yes	Yes	Yes	Yes	Yes	No	Yes	Yes
Petit et al. [28]	This study is not randomized. Therefore, COCHRANE guidelines do not apply here.									
Khoo et al. [27]	**Yes**	**Yes**	**No**	No	Yes	Yes	Yes	**Yes**	**Nr**	**Yes**
Guo et al. [25]	**Yes**	**Yes**	**No**	No	Yes	Yes	Yes	**No**	**Yes**	**Yes**
Yan et al. [30]	**Yes**	**Yes**	**Yes**	Yes	Yes	Yes	Yes	**No**	**Yes**	**Yes**
Mullins et al. [17]	**Yes**	**No**	**Yes**	Yes	No	Yes	Yes	**No**	**No**	**Yes**
Gejl et al. [31]	**Yes**	**No**	**Yes**	Yes	Yes	Yes	Yes	**No**	**No**	**Yes**
Gejl et al. [32]	**Yes**	**No**	**Yes**	Yes	Yes	Yes	Yes	**No**	**NR**	**Yes**
Watson et al. [33]	**Yes**	**Yes**	**Yes**	Yes	No	Yes	Yes	**No**	**No**	**Yes**
Kahal et al. [35]	This study is not randomized. Therefore, COCHRANE guidelines do not apply here.									
Moulton et al. [36]	This study is not randomized. Therefore, COCHRANE guidelines do not apply here.									
Grant et al. [34]	This study is not randomized. Therefore, COCHRANE guidelines do not apply here.									
